# Is the tea or teacup good? The effect of visual and haptic sensory processing of teacups on the perception of tea flavor

**DOI:** 10.3389/fpsyg.2024.1427665

**Published:** 2024-07-23

**Authors:** Su-Chiu Yang, Li-Chieh Hsu

**Affiliations:** School of Arts and Design, Sanming University, Sanming, China

**Keywords:** tea culture, visual, tactile, tea perception, intermediary effect

## Abstract

Prior research on the relationship between the taste, aroma and drinking utensils of beverages tends to focus on topics such as alcohol, sparkling beverages, juice, coffee, and hot chocolate. There is limited research focused on the interdependence between the perception of teacups and the tea taste. The literature has not yet found any research covering the impact of visual shape and the tactile sensation of teacups on the perception of tea flavor. Therefore, this study proposed six hypotheses related to the teacup shape and texture, teacup preference and taste and smell of tea. This study involved experimental design and questionnaire data collection, using a convenience sampling method to recruit 102 participants voluntarily. The research results are: (1) Age and gender have an impact on the taste and aroma perception of tea; (2) The width, height, rim thickness and smoothness of the teacup surface do have an impact on the perception of taste and fragrance of tea. (3) The preference of teacup played an intermediary effect between tea taste and the shape and texture of teacup. The implications of these findings on the perception of tea flavor are discussed.

## 1 Introduction

According to Joshi (2023), writing in Tea Statistics, in 2023, more than 87% of Millennials drank tea, resulting in 4 out of 5 tea consumers and 3 billion cups of tea being consumed around the globe every day. Despite the global popularity and consumption figures for tea, there has been remarkably little research on the tea drinking vessel compared to other beverages, such as coffee, hot chocolate, cola, etc. (Piqueras-Fiszman et al., 2012; Van Doorn et al., 2014, 2017; Spence and Wan, 2015; Cavazana et al., 2017). These beverages were consumed from a wide range of different receptacles, that vary in terms of their size, texture, shape, material properties, color, etc. The old saying, “you eat with your eyes” (Delwiche, 2012) reveals the truth that our experience of eating food or drinking beverage is more than just a chemical reaction between the food and our sensation (Spence and Piqueras-Fiszman, 2014; Spence, 2017; Risso et al., 2019). It is the context of the stimuli and the complex reactions among the different sensory aspects, as well as the brain integrating them into a whole experience. The process by which human sensory receptors receive external stimulation does not operate independently, but is compounded and performed simultaneously. Research on visual effects on food taste includes the effects of food color, food texture, and eating environment on the “taste” and “aroma” of food (Hogenkamp et al., 2011; Woods et al., 2011; Piqueras-Fiszman et al., 2012; Stewart and Goss, 2013). Therefore, when we eat something, in addition to smell and taste, sensory stimulation such as vision, hearing, and touch also contribute to the formation of the “flavor system” (Small et al., 2004; Shepherd, 2006). A number of studies had pointed out that, in addition to the texture and appearance of the food itself affecting the taste and aroma of the food, the visual stimulation of the containers holding food or drinks in restaurants or supermarkets is closely related to the enjoyment and charm of the food (Shepherd, 2006; Zellner et al., 2010, 2011; Mielby et al., 2012; Piqueras-Fiszman et al., 2013). Hence, over the past two decades, many studies began to investigate these important aspects. The study by Piqueras-Fiszman et al. (2012) showed that the color of food and drink containers can enhance the flavor and aroma components. The study found that subjects had different perceptions of sweetness and flavor for the same hot chocolate while they were using the different color of cups.

A number of studies (Madden et al., 2000; Piqueras-Fiszman et al., 2012; Wan et al., 2014, 2015) focusing on coffee, juice, or wine have shown that the visual shape of beverage utensils affects the judgment of drinks with a specific color, as well as the expectations for the aroma of drinks. For example, if a purple drink is placed in a wine glass, it will be considered as wine, while if a purple drink is placed in a straight glass, it will be considered as grape juice. Li et al. (2020) conducted a cross-cultural study on the impact of the visual appearance of the container on the subjective evaluation and taste expectations of tea by using 1,100 Chinese and 100 Americans as subjects. They displayed on-line photos of Chinese or British tea sets containing Chinese and British brands of green tea to explore how the subjects felt about each cup of tea and to evaluate their taste expectations. The study found that the tea set affected bitterness expectations for Chinese subjects with the Chinese brand of green tea and enjoyment expectations for the tea.

The research by Van Doorn et al. (2014) showed that the appearance color of the cup affected the subjects’ sensory perception of the intensity of the same coffee. Researchers found that the same coffee in white ceramic mug was perceived as having a richer taste than coffee in a transparent mug. Apart from the perception of taste, the color of the cup also affects the perception of coffee temperature (Guéguen and Jacob, 2012) and the judgment of the taste of regular water and sparkling water (Risso et al., 2015).

Visual and tactile stimuli have been shown to alter the perception of taste, smell and flavor. Studies tested whether how subjects saw and touched the wine glass affected the aroma of wine. The results showed that when the subjects could not see or touch the wine glass, the shape of the wine glass had little impact on the perception of the aroma of the wine. On the contrary, if the subject sees and holds the wine glass, the shape of the wine glass has a considerable impact on the perception of the aroma of the wine (Cliff, 2001; Delwiche and Pelchat, 2002; Russell et al., 2005). In addition, some studies have found that even experienced wine tasters would still be affected by the shape of the wine glass in their perception of aroma (Vilanova et al., 2008). A similar research conclusion was reached by Peng and Yang (2017), indicating that the shape of the teacup affects the tea taste. Even experienced professional tea reviewers gave different ratings to the aroma and taste of tea brewed from the same tea at the same time and in different shapes of teacups. Van Doorn et al. (2017) conducted a cross-cultural study on how the shape of coffee cups affects the taste of coffee in China, Colombia and the United Kingdom. They found that visual information such as the diameter of the coffee cup and the height of the cup is closely related to consumers’ sensory expectations. Besides the visual elements affecting the taste perception of beverages, the research also found that the taste-tactile multisensory interactions affected beverage perception. Schifferstein (2009) conducted a study by using five cups that were almost identical in shape and size but made from different materials, to have participants rate the empty cups and evaluate the experience of drinking hot tea or a chilled soft drink from these cups. The results found that the drinking experience was linked to the cups. In the study of tactile aesthetics, Kreifeldt (2013) proposed that the visual image of the appearance of an object can evoke appropriate tactile feelings and produce visual and tactile cross-module aesthetics. Therefore, whether the imagined tactile sensations and actual tactile sensations evoked by the visual design of beverage vessels also have a cross-modal impact on the smell and taste of beverages arose researchers’ curiosity.

Tea is the most widely consumed beverage globally. In 2022, China was the largest tea consumption country in the world, accounting for more than 41% of global tea consumption (China Association for the Promotion of International Agricultural Cooperation, 2022). The study by Spence and Wan (2016) specifically pointed out that, although there are a large number of tea drinkers around the world, there is little research on the issue of suitable utensils for tea drinks, and especially a lack of in-depth investigation and research on Chinese teacups. To summarize, the available literature indicates that various visual and tactile factors in drinking utensils design could be expected to have effects on a beverage, and in particular that color, shape, and texture are key aspects to consider. As a whole, however, research on the relation between perceived quality and consumer perception of tea specifically is limited. In particular, there is a gap in research on the impact of visual and tactile sensations on tea flavor perception. This research will therefore address the visual and tactile effect of teacups on consumer perception of tea quality within the theoretical motivation of the “flavor system” (Shepherd, 2006) context and uses an experimental survey and questionnaires to collect data.

## 2 Materials and methods

### 2.1 Research hypothesis

Visual and tactile factors are key aspects in this study, therefore, based on the previous literature related to the impacts of the visual shape and texture of the utensils on the taste perception of tea or other beverages, research hypotheses were proposed based on the following description. First, for the visual part, according to Li et al. (2020) research, the perception of differences in the visual shapes of Chinese and Western teacups and utensils of different shapes affects subjects’ expectations for the taste of tea. Other related literature mostly focused research on wine, coffee, hot chocolate or juice, etc. The shape of the wine glass had an impact on the aroma of wine (Delwiche and Pelchat, 2002) and the “cup diameter” and “cup height” affected the characteristic aroma, bitterness, intensity and sweetness of coffee (Van Doorn et al., 2017). Even Cavazana et al. (2017) found that cola in a typical cola cup feels sweeter and more intense than cola in a water glass or a plastic cup. Therefore, the shape differences of comprehensive utensils is hypothesized to have impacts on the taste perception of tea and the following three hypotheses are made:

**H1**: The width difference of the teacup affects the flavor of the tea.

**H2**: The height difference of the teacup affects the flavor of the tea.

**H3**: The thickness of the teacup’s rim affects the flavor of the tea.

Secondly, the touch of hand, skin and lips were the main receptors stimulated in this study. Delwiche and Pelchat (2002) conducted a blind taste study and found that professional tasters were influenced by the visual image of the glass when judging taste and aroma. Other related literature showed that both professionals and amateurs were inevitably going to be affected by touch when tasting tea or wine (Vilanova et al., 2008; Peng and Yang, 2017). Regarding the impact of surface texture on the nasal smell and oral sensation of the wine tasting experience, Wang and Spence (2018) found that when subjects touched wine glasses with contrasting textures of velvet and sandpaper, the wine in the velvet glass had a more fruity aroma than the rough wine glass, and the taste was also sweeter and more pleasant. Schifferstein (2009) found that the materials of the utensil and the touch of the skin have a significant impact on the sensory attributes “warmth” and “sweetness” of hot Earl Gray tea or ice lemon drinks and an impact on the bitterness of coffee and the sweetness of chocolate (Van Rompay et al., 2017). Tu et al. (2015) took Chinese tea drinks as the research object and found that utensils with different tactility had an impact on the sweetness and coldness of the tea drinks. The external information of the texture of teacup is integrated with information from taste receptors through the perception of other modalities. The fourth hypothesis is made as follows:

**H4**: The smoothness of the teacup surface affects the flavor of the tea.

Besides the above external information, internal emotional state may also influence taste perception through awareness. Prior studies had shown that emotional states influence taste perception. Most studies found negative emotion tended to exert the opposing effect on taste perception (Noel and Dando, 2015; Wang and Spence, 2016, 2018; Reinoso-Carvalho et al., 2019, 2020; Zushi et al., 2023). Other research found that there is a metaphorical association between emotions and taste, i.e., sweetness is related to positive emotion (Liang et al., 2020; Zhou and Tse, 2020). The shape and texture of a teacup are the determinants of the consumer’s preference when choosing teacups. Therefore, teacup preference should be classified as an emotional response. The fifth and sixth hypotheses are made as follows:

H5: The shape and texture of teacup affects the preference of teacup.

H6: The preference of the teacup shape affects the flavor of the tea.

**Figure 1:**
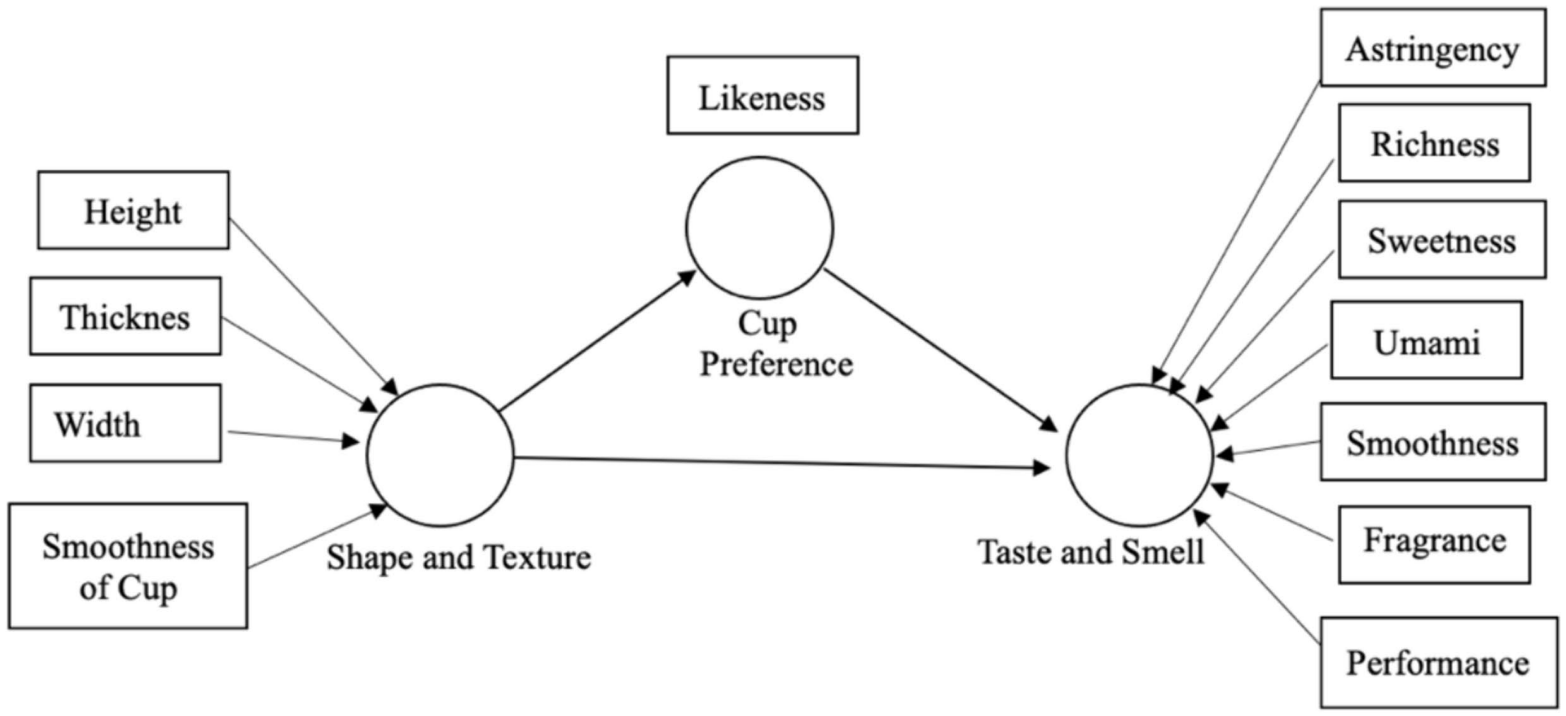
Relationship between teacup shape and texture, teacup preference and the tea taste and smell.

The relationship model of teacup shape and texture, teacup preference and the tea taste and smell is shown in Figure 1.

### 2.2 Experimental materials

The visual shape and texture of the teacup are the independent variables in this study. The dependent variables are the taste, fragrance and overall performance of the tea. Chinese famous high mountain tea, Dahongpao, was selected as the experimental material for this study. A white porcelain evaluation cup with a capacity of 150 mL was used to hold 3 grams of tea. It was brewed with 100°C water and soaked for 5 min. According to expert evaluation standards, the flavor of tea drinks is classified into (a) the intensity of tea astringency; (b) the intensity of tea umami; (c) the intensity of tea sweetness; (d) the intensity of tea smoothness; (e) the intensity of tea richness and (f) degree of tea fragrance. This study included the overall appreciation of tea and the degree of preference for teacups, then designed a questionnaire based on the 5-point Likert scale.

### 2.3 Experimental products

**Figure 2:**
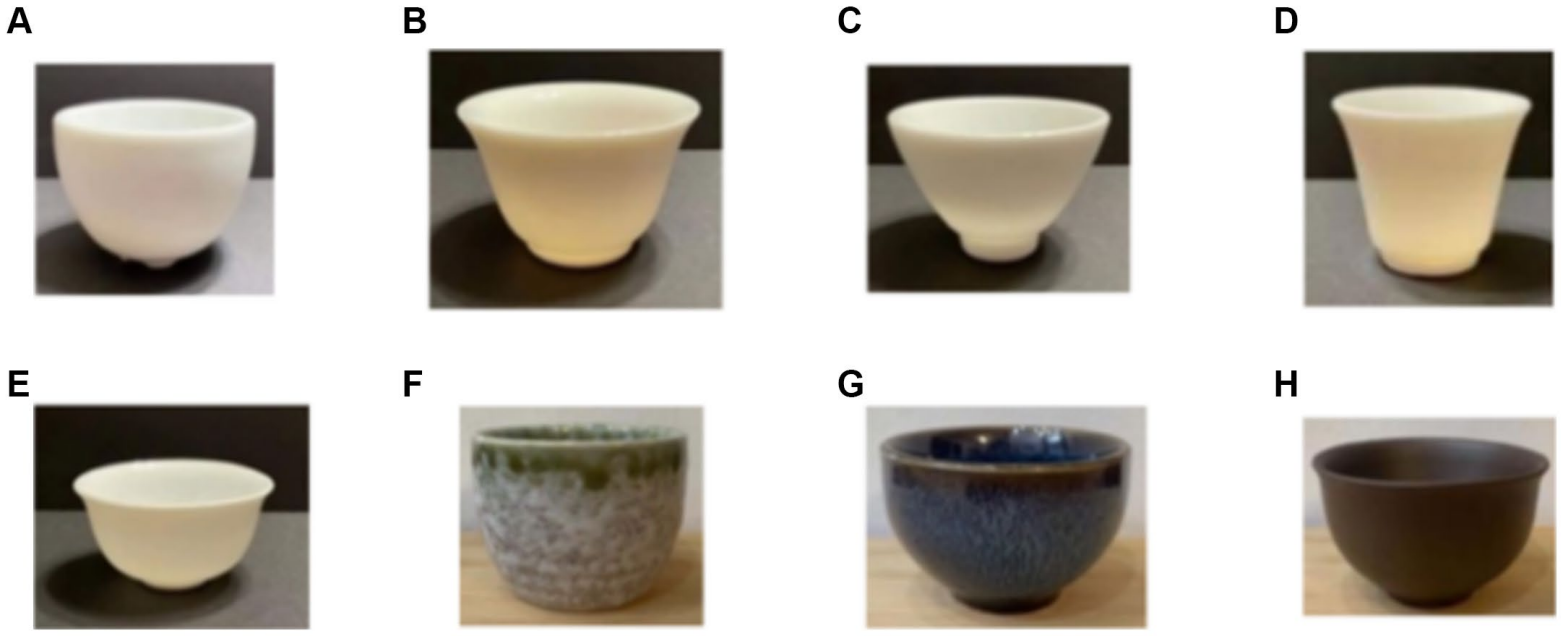
Cup A-H.

Eight teacups with similar capacities but different shapes and textures were selected as materials for this study (Figure 2). The description of the eight cups are as follows: (A) Cup A with a wide mouth and shallow body (cup diameter width: 7.7 cm, cup height: 3.8 cm, cup rim thickness: 0.4 cm); (B) Cup B with outward wide a mouth and shallow body (cup diameter width: 6.7 cm, cup height: 3.5 cm, cup rim thickness: 0.3 cm); (C) Cup C with an open mouth and a narrow shallow body (cup width: 5.6 cm, cup height: 5.6 cm, cup rim thickness: 0.4 cm); (D) Cup D with a narrow deep body (cup diameter width: 4.7 cm, cup height: 6.0 cm, cup rim thickness: 0.25 cm); (E) Cup E with an open narrow shallow body (cup diameter width: 6.4 cm, cup height: 3 cm, cup rim thickness: 0.2 cm); (F) Cup F with a narrow mouth, deep body and protruding surface (cup diameter width: 5.2 cm, cup height: 4.8 cm; cup rim thickness: 0.5 cm); (G) Cup G with a wide mouth, shallow body and smooth surface (diameter width: 6.5 cm, cup height: 4.2 cm, cup rim thickness: 0.6 cm); (H) Cup H with rough surface, outward wide mouth and shallow body (cup diameter width: 7 cm, cup height: 3.8 cm, cup rim thickness: 0.25 cm).

### 2.4 Participants

The professional classification of the tea flavor been described in section 2.3. It is hard for the novices or general consumer to distinguish between tea taste and fragrance according to the professional classification. In order to achieve the desired accuracy, validity and bias avoidance of the experiment for this study, therefore, participants were mainly recruited through the members of relevant tea associations or organizations or volunteers who are interested in this study recruited from the community. Therefore, participants covered everyday tea drinkers, professional tea lovers as well as novices or general consumers. One hundred and two participants took part in this study. The basic information of the subjects such as gender, age, education and frequency of tea drinking is summarized in Table 1. The sample comprised 66 females and 36 males. Their ages were mainly distributed between 45 and 64 years old, accounting for 68%. All of the participants have at least a bachelor’s degree, and 59% of the participants drink tea every day.

**Table 1:** Participant demographics.

Variable		Percentage (*N* = 102)	Variable		Percentage (*N* = 102)
Gender	Male	35%	Age	15–24	6%
	Female	65%		25–34	14%
Education	University	62%		35–44	12%
	Post-Graduate	38%		45–54	23%
Frequency of drrinking tea	Everyday	59%		55–64	40%
	Occasionally	41%		Above 65	5%

This study collected the physiological and psychological perception of the subjects during the tea drinking process and did not involve the use of human specimens for biological behavior, physiology, psychology, genetics, medicine and other research. According to the National Taiwan Normal University Research Ethics Review Committee’s exemption case-project host self-evaluation system, the subjects in this study were all adults, and they all accepted the test freely and without coercion, and this study was exempt from ethical review by the ethical review committee. The participants provided their written informed consent to participate in this study.

### 2.5 Procedure

Participants were informed in advance to rinse their mouths before taking the test to ensure that there were no residues in the mouth. The process of the experiment and the contents of the questionnaire were explained in advance. After that, the researcher gave the subjects 10 min to fill out their basic personal information and read the reference information of the questionnaire. To prevent the tea from getting cold, the tea was brewed within these 10 min, poured into a teacup, and placed in front of the subject. The subject drank the tea in the given teacup in order. Then, they made subjective judgments about the taste and aroma, and filled in the judgment results in the second part of the questionnaire.

### 2.6 Data analysis

SPSS quantitative statistical analysis was used to examine the correlation between variables. Single-factor variation was used to analyze the differences in flavor of tea drinks between the two groups and their correlation with other variables. Pearson’s product coefficient correlation detected whether there is an interaction or correlation between the taste of tea, the aroma of tea and teacup preference. The independent-sample T-test was used to detect whether gender differences in the subject group had an impact on the taste of the tea. For more than three variables, analysis of variance (ANOVA) was applied to determine whether the differences between groups reached a significant level.

## 3 Results

### 3.1 Tea taste data from the 102 participants

Tea taste data from the one hundred an two subjects were analyzed. The descriptive statistical analysis found the average number of cup A was the highest in umami, smoothness and fragrance of tea taste perception. Cup B presented the highest average number in sweetness of tea taste perception. Regarding the overall performance of tea taste and teacup preference, cup D presented the highest average number. Cup F presented the highest average number in astringency and richness of tea taste perception. In terms of the appearance characteristics of the teacup, cup A, B and D are white and have a smooth surface, while the surface of cup F is smooth but granular. Conclusively, Cup A and B with the common characteristic of a wide cup mouth had a higher score on the umami, smoothness and sweetness which is found on the front part of our tongue taste map. Cup F with the characteristic of thickest cup rim and convex surface presented the highest average number in astringency and richness of tea perception which are on the inner side of the tongue taste map.

### 3.2 Independent sample T-test

Independent sample T-tests of SPSS were employed to test to the influence of gender differences on the perception of tea taste and fragrance. Results (Table 2) revealed that there were significant differences only in the perception astringency and richness of tea taste. Male participants’ perception of the astringency and richness of the tea taste was significantly higher than that of female participants.

**Table 2:** Comparison of gender differences in the flavor of tea.

variable	Gender	Mean	Standard deviation	T-value
tea astringency	Male	3.41	1.078	−5.649**
	Female	2.99	.948	
tea richness	Male	3.43	.908	−7.676**
	Female	2.89	1.033	

**p < 0.01.

### 3.3 Analysis of variance

ANOVA was used to test the impact of age differences on the taste of tea. It was found that the age variables (Table 3) had significant differences in the taste, smell and overall performance of tea. Tukey HSD multiple comparisons were used to analyze the differences in the impact of different age groups on the flavor of tea.

**Table 3:** ANOVA analysis of differences in flavor compared to age.

Variable	Source	Sum of squares	Freedom	Mean square	F-test	Significance
Astringency	Between groups	63.049	5	12.610	12.225	<0.001^***^
	Within groups	835.495	810	1.031		
	Total	898.544	815			
Umami	Between groups	83.624	5	16.725	19.396	<0.001^***^
	Within groups	698.450	810	0.862		
	Total	782.074	815			
Sweetness	Between groups	43.968	5	8.794	8.584	<0.001^***^
	Within groups	829.738	810	1.024		
	Total	873.706	815			
Smoothness	Between groups	42.750	5	8.550	8.622	<0.001^***^
	Within groups	803.206	810	0.952		
	Total	845.956	815			
Richness	Between groups	79.119	5	15.824	16.615	<0.001^***^
	Within groups	771.425	810	0.952		
	Total	850.544	815			
Fragrance	Between groups	17.058	5	3.502	4.352	<0.001^***^
	Within groups	651.669	810	0.085		
	Total	669.176	815			
Overall performance	Between groups	31.739	5	6.348	6.658	<.001***
	Within groups	772.250	810	0.953		
	Total	803.989	815			

^***^p < 0.001.

Table 4 showed that the younger the subjects, the stronger the astringent, rich and better overall performance of tea taste; while the older the subjects, the stronger the umami, sweet, smooth taste and better fragrance of tea taste. It could be interpreted that younger subjects, who tend to be affected by the tea beverage on the market, have less a traditional tea drinking experience than older subjects, so they might be more sensitive to the astringent and rich perception of tea taste. On the contrary, the older subjects have more tea drinking experiences, so they are more sensitive to the sense of umami, sweetness, smoothness and fragrance of tea taste. Therefore,we concluded that age differences reflect the perception of different flavor and fragrance of tea.

**Table 4:** Multiple comparative analysis of Tukey HSD.

Dependent variable	Age (I)	Age (J)	Mean difference	Standard error	Significance	95% Confidence interval	
						Lower limit	Upper limit
Astringency	15–24	35–44	0.906^***^	0.180	0.000	0.39	1.41
		Above 64	1.188^***^	0.254	0.000	0.46	1.91
	25–34	35–44	0.869^***^	0.243	0.000	0.17	1.57
		55–64	0.377^**^	0.206	0.006	0.07	.68
		Above 64	1.150^**^	0.396	0.000	0.01	2.29
	35–44	45–54	−0.531^**^	0.134	0.001	−0.91	−0.15
		55–64	−0.492^***^	0.116	0.000	−0.82	−0.16
Umami	15–24	25–34	−0.700^***^	0.159	0.000	−1.15	−0.25
		35–44	−1.281^***^	0.286	0.000	−2.10	−0.46
		45–54	−0.979^***^	0.155	0.000	−1.42	−0.54
		55–64	−1.203^**^	0.142	0.000	−1.61	−0.80
		Above 64	−0.750^*^	0.232	0.016	−1.41	−0.09
	25–34	35–44	−0.581^***^	0.127	0.000	−0.94	−0.22
		55–64	−0.503^**^	0.097	0.000	−0.78	−0.23
Sweetness	15–24	25–34	−0.663^**^	0.173	0.002	−1.16	−0.17
		35–44	−1.031^***^	0.179	0.000	−1.54	−0.52
		45–54	−0.792^***^	0.169	0.000	−1.27	−0.31
		55–64	−0.922^***^	0.155	0.000	−1.36	−0.48
		Above 64	−0.937^**^	0.253	0.003	−1.66	−0.21
Smoothness	15–24	35–44	−0.750^***^	0.176	0.000	−1.25	−0.25
		55–64	−0.641^***^	0.152	0.000	−1.08	−0.21
	35–44	45–54	0.583^***^	0.131	0.000	0.21	0.96
	45–54	55–64	−0.474^***^	0.097	0.000	−0.75	−0.20
Richness	15–24	25–34	0.788^***^	0.167	0.000	0.31	1.26
		35–44	1.406^***^	0.173	0.000	0.91	1.90
		45–54	1.229^***^	0.163	0.000	0.76	1.69
		55–64	.922^***^	0149	0.000	0.50	1.35
		Above 64	1.188^***^	0.244	0.000	0.49	1.88
	25–34	35–44	0.619^***^	0.134	0.000	0.24	1.00
		45–54	0.442^**^	0.121	0.004	0.10	0.79
	35–44	55–64	−0.484^***^	0.111	0.000	−0.80	−0.17
	45–54	55–64	−0.307^*^	0.095	0.017	−0.58	−0.03
Fragrance	25–34	45–54	−0.446^**^	0.111	0.001	−0.76	−0.13
		55–64	−0.284^*^	0.094	0.030	−0.55	−0.02
	35–44	45–54	−0.396^*^	0.118	0.011	−0.73	−0.06
Overall performance	15–24	25–34	0.688^**^	0.167	0.001	0.21	1.16
		45–54	0.521^*^	0.163	0.018	0.06	0.99
		Above 64	1.06^***^	0.244	0.000	0.37	1.76
	25–34	55–64	−0.359^**^	0.102	0.006	−0.65	−0.07

* *p* < 0.05,

** *p* < 0.01,

*** *p* < 0.001.

### 3.4 Pearson’s product coefficient correlation

The flavor and fragrance of tea do not exist as a single entity. Normally due to the production process, the flavor and fragrance are mixed into the tea and present a different level of perception in taste and smell. In order to understand whether the taste of tea, the fragrance of tea and teacup preference interact with each other, Pearson’s product coefficient correlation was used to identify the correlation between variables and the square value of the product coefficient (R^2^) was used as the determining coefficient to display the percentage of explanation between variables (Table 5). The coefficient of determination R^2^ between 0.16 and 0.47 means a moderate correlation between variables. Results showed that: (1) The astringency of the tea taste had a moderate correlation with the richness of tea taste; (2) The umami of tea taste had a moderate correlation with the sweetness and smoothness of the tea taste; (3) The sweetness of tea taste had a moderate correlation with the umami and smoothness of the tea taste; (4) The overall performance of the tea taste had a moderate correlation with the umami, sweetness, smoothness and fragrance of the tea taste; (5) Although there was a correlation between the preference of the teacup and the taste and richness, fragrance and the overall performance of tea, the correlation was low (R^2^ < 0.16).

**Table 5:** Correlation matrix between tea flavor and teacup preference.

	Astringency	Umami	Sweetness	Smoothness	Richness	Fragrance	Performance	Cup preference
Astringency	1	−0.011	−0.19	0.029	0.508**	0.188^*^	0.237^**^	−0.014
					(R^2^ = 0.258)	(R^2^ = 0.035)	(R^2^ = 0.056)	
Umami	−0.011	1	0.613**	0.569**	0.057	0.266^**^	0.464**	0.098
			(R^2^ = 0.376)	(R^2^ = 0.324)		(R^2^ = 0.071)	(R^2^ = 0.215)	
Sweetness	−0.19	0.613**	1	0.657**	0.134**	0.255^**^	0.409**	0.078
		(R^2^ = 0.376)		(R^2^ = 0.432)	(R^2^ = 0.018)	(R^2^ = 0.065)	(R^2^ = 0.167)	
Smoothness	0.029	0.569**	0.657**	1	0.255^**^	0.343^**^	0.432**	0.149
		(R^2^ = 0.324)	(R^2^ = 0.432)		(R^2^ = 0.065)	(R^2^ = 0.118)	(R^2^ = 0.187)	
Richness	0.508**	0.057	0.134	0.255^**^	1	0.296^**^	0.323^**^	0.166^*^
	(R^2^ = 0.258)			(R^2^ = 0.065)		(R^2^ = 0.088)	(R^2^ = 0.104)	(R^2^ = 0.028)
Fragrance	0.188^*^	0.266^**^	0.255^**^	0.343^**^	0.296^**^	1	0.391^**^	0.164^**^
	(R^2^ = 0.035)	(R^2^ = 0.071)	(R^2^ = 0.065)	(R^2^ = 0.118)	(R^2^ = 0.088)		(R^2^ = 0.153)	(R^2^ = 0.029)
Performance	0.237^**^	0.464**	0.409**	0.432**	0.323^**^	0.391**	1	0.280^**^
	(R^2^ = 0.056)	(R^2^ = 0.215)	(R^2^ = 0.167)	(R^2^ = 0.187)	(R^2^ = 0.104)	(R^2^ = 0.153)		(R^2^ = 0.078)
Cup preference	−0.014	0.098	0.078	0.149	0.166^*^	0.164^**^	0.280^**^	1
					(R^2^ = 0.028)	(R^2^ = 0.029)	(R^2^ = 0.078)	

^*^p < 0.05, ^**^p < 0.01. Bold value indicates the coefficient of determination R^2^ between 0.16 and 0.47 means a moderate correlation between variables.

Pearson’s product coefficient correlation is also used to examine the influences of cup shape and texture on the flavor perception of tea. Table 6 revealed that the texture of the cup surface has impacts on the astringency, umami, sweetness, smoothness, fragrance, performance of tea taste perception and the cup preference, yet the correlation was low (R^2^ < 0.16).

**Table 6:** Correlation matrix teacup shape and texture and flavor, teacup preference.

	Smoothness of cup surface	Cup height	Cup thickness	Cup width
Astringency	−0.122^**^ (R^2^ = 0.015)	0.062	0.017	−0.071^*^ (R^2^ = 0.005)
Umami	0.212^**^ (R^2^ = 0.045)	−0.005	−0.001	−.0.108
Sweetness	0.134	0.048	−0.030	−0.024
Smoothness	0.130^**^	0.040	0.043	−0.022
Richness	0.110^**^ (R^2^ = 0.012)	−0.033	0.006	0.026
Fragrance	0.006	−0.076^*^ (R^2^ = 0.006)	0.036	0.116^**^ (R^2^ = 0.013)
Performance	0.184^*^ (R^2^ = 0.034)	0.083^*^ (R^2^ = 0.007)	0.050	−0.067
Cup Preference	0.117^**^ (R^2^ = 0.013)	0.095^*^ (R^2^ = 0.009)	0.067	−0.066

^*^p < 0.05, ^**^p < 0.01. Bold value indicates the coefficient of determination R^2^. R^2^ < 0.16 means a low correlation between variables.

### 3.5 SEM and PL-SEM

**Figure 3:**
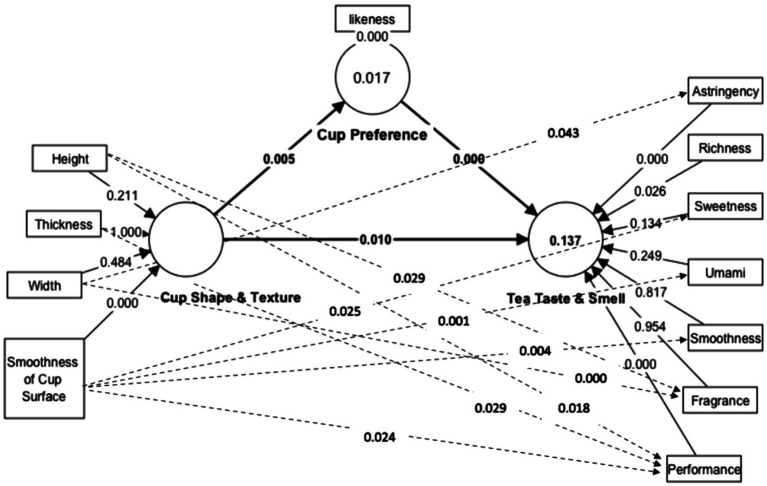
Findings.

In the field of social science, many implicit behaviors and psychological traits cannot be observed and measured, and these are latent variables that exist between explicit variables. Therefore, structural equations model (SEM) uses a regression method to estimate the causal relationship between measured variables (Wu, 2013, p. 426). In this study, the non-duality of teacup appearance tea led to differences in visual and tactile sensory perception and had a complex relationship with the taste, fragrance and overall performance of the tea taste. Therefore, the proposed model (Figure 1) was tested using Smart PLS and multiple regression. The effect-cause relationship path diagram was drawn as Figure 3. Smart PLS results were shown on the solid line in Figure 3. Path coefficient indicated that the model of cause-effect relation between tea taste and the shape and texture of teacup was significant (*p*-value is 0.010 < 0.05). It also indicated that the relation between tea taste and the preference of teacup (*p*-value is 0.000) and the relation between the shape and texture of teacup and the preference of teacup (*p*-value is 0.005) were salient. We used multiple regression (the dotted line in Figure 3) to explore the impacts of all the independent variables of teacup shape and texture on the flavor of tea. The results showed that the height of teacup had significant influence on the smell (*p*-value is 0.029) and overall performance of tea (*p*-value is 0.018). The thickness of teacup rim influenced the overall performance (*p*-value is 0.029) of tea significantly. The width of teacup showed a significant effect on the astringency (*p*-value is 0.043) and the smell of tea (*p*-value is 0.000). The surface smoothness of the teacup affected the smoothness (*p*-value is 0.004), umami (*p*-value is 0.001), sweetness (*p*-value is 0.025) and the overall performance (*p*-value is 0.024) significantly. In all, the surface texture of teacup is the most influential factor on the taste of tea.

## 4 Discussion

From the results of Pearson’s product coefficient correlation and PLS-SEM, it was found that the visual shape and the hand-touch feelings of the teacup had influences on the preference of teacup, as well as the taste and smell of the tea. At the same time, the preference of teacups also affects the taste and smell of the tea. Figure 3 showed that the teacup preference is the independent variable which influences the tea taste perception while the shape and texture influence the perception of tea taste. The preference of teacup plays an intermediary effect (as presented in Figure 1) between tea taste and the shape and texture of the teacup. So, the hypotheses H1 to H6 were valid. Both external information of the shape and texture of teacup and internal information of the teacup preference had influences on the perception of tea taste.

Visually, the width, height, and thickness of the rim of the teacup affect the taste, fragrance and overall performance of the tea. Relevant literature (Van Doorn et al., 2017; Cliceri et al., 2018; Yang et al., 2019; Yang and Peng, 2021) showed that the flavor of coffee, beverages and tea is related to the height of the vessel. A higher height of the teacup means that the color of the tea will be darker. The cross-modal correspondence between vision and taste may affect the intensity of the tea. Therefore, the results of integrating the diameter width, height of the teacup and the thickness of the teacup rim affecting the taste, fragrance and overall performance of the tea are consistent with the research of Van Doorn et al. (2017), which involved “cup diameter,” “cup height” and “cup thickness” with coffee flavor expectations.

Van Doorn et al. (2017) found that “cup diameter” and “cup height” can affect the aroma, bitterness, intensity and sweetness of coffee, but “cup thickness” only affects the temperature of coffee. However, this present study found that “teacup thickness” had impact on the flavor of tea. Normally, cups with a thick rim are also thicker on the overall cup shape. Although there is a significant difference in size between Chinese teacups and western coffee cups, the thickness of the Chinese teacup has a thermal insulation effect on maintaining the temperature of the tea as well as better expressing the taste, fragrance and overall performance of Chinese tea.

In this study, it was concluded that the width, height, the rim thickness and the smoothness surface of the teacup do have impacts on the performance of the tea. Chinese tea is always served hot. The standard procedure of drinking Chinese tea is to observe the color of the tea first, then smell the aroma of the tea, and taste the tea last. Therefore, Table 7 showed that cup A with a thick mouth and cup B with a wide mouth performed better in the umami, sweetness, smoothness and fragrance of tea taste. For a consumer, the high cup D may be difficult to observe the color of the tea and smell it unfavorably, but the shape of cup D is more likeable and has better overall performance of tea taste. The visual system preattentively extracts pattern features might help to predict the haptic qualities of surfaces, such as grain size, density, or regularity (see Di Stefano and Spence, 2022, for a multisensory review on roughness). Therefore, cup F, with a deep and protruding ventral surface, appears as visually rough and performed better on astringency and richness taste of tea in line with the findings of Carvalho et al. (2020). Carvalho et al. found that rough coffee cups enhanced professional subjects’ perception of the original sour taste of specialty coffee. It can be inferred that participants drank tea with a teacup having a non-smooth surface, the sensory transfer intensified the astringent taste of the tea, resulting in a strong irritation due to the intensity of the taste of tea in Chinese tea products sometimes being interpreted as a strong taste or astringency (Tea Industry Improvement Center of the Council of Agriculture, Executive Yuan, 2001). Therefore, cup F with a smooth, protruding surface and deep body has an amplifying and strengthening effect on other taste sensations of the tea due to sensory transfer, such as the astringency and richness of taste.

**Table 7:** The percentage of measurement results of (A–E) teacup and tea performance by the subject.

Tea variable	Cup variable		f/total	pot	Tea variable	Cup variable		f/total	pot
Astringency	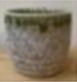	F	23/102	23	Richness	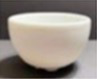	F	41/102	41
Umami	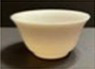	A	59/102	58	Fragrance	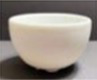	A	84/102	82
Sweetness	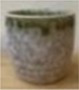	B	51/102	50	Performance	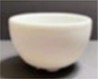	D	54/102	53
Smoothness	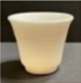	A	42/102	41	Cup preference	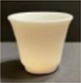	D	84/102	82

## 5 Conclusions and suggestions

Results of this study showed that hypotheses 1 to 6 were valid, demonstrating the height, width, thickness and smoothness of the teacups had effects on the flavor of the tea perception, the shape and texture of teacup influenced the preference of teacup, and the preference of teacup had an effect on the flavor of the tea perception. It showed that the teacup preference is the independent variable which influences the tea taste perception while the shape and texture influence the tea taste perception. Teacup preference played an intermediary effect (as presented in Figure 1) between tea taste and the shape and texture of teacup. In other words, that is in line with prior studies that consumers’ emotions might effect taste perception (Noel and Dando, 2015; Carvalho et al., 2020; Zushi et al., 2023). Therefore, both external information regarding the shape and texture of teacup and internal information of the teacup preference influenced the perception of tea taste.

Since the shape and texture of the teacup affects the preference and the perception of tea taste, the research results are of strong commercial value. From a marketing perspective, it suggests considering choosing appropriate teacups for specific consumers to increase the sales of tea. Combing the results of age and gender factors, male subjects have significantly stronger feelings about the astringency and richness of tea than female subjects. Therefore, for general female consumers, a narrow mouth and deep body and a smooth pattern on the surface of teacup, like cup F, is recommended because this kind of teacup can enable ordinary female consumers to increase the astringency and richness of the tea during the tea tasting process. For younger consumers, who have a weaker sense of the umami, sweetness and smoothness of the tea taste, it is suggested to choose a wide mouth cup (like cup A) or a wide mouth with shallow body (like cup B) to increase the freshness, sweetness and smoothness of the taste of the tea.

There are a variety of teas, each with their own unique characteristics. Moreover, tea is a subjective drink, and preferences vary from person to person. Therefore, the findings of this study can provide tea sellers, tea manufacturers or tea consumers with a basis for choice when brewing tea. According to different tea characteristics or different consumers’ preferences, these findings can provide suggestions for choosing a suitable teacup to appropriately express the characteristics of the tea.

## Data availability statement

The original contributions presented in the study are included in the article/supplementary material, further inquiries can be directed to the corresponding author.

## Ethics statement

This study used the National Taiwan Normal University Research Ethics Review Board's self-assessment system for project supervisors. This study uses the physiological and psychological reaction data of the subjects during the tea drinking process to obtain, investigate, and analyze, but does not involve the use of human specimens for biological behavior, physiology, psychology, genetics, medicine, etc. research. The subjects of this study are all adults, and the subjects are not interns, aboriginals, pregnant women, people with physical or mental disabilities, or mentally ill people. All subjects accepted the test freely and without coercion. And this research conforms to "a non-named, non-interactive and non-invasive research conducted in a public place, and no specific individual can be identified from the information collected", "the use of information that has been legally disclosed to the public, and the use of the information complies with its disclosure "well-known purpose" and "the research project is of the lowest risk, and the risk to the research subjects is no higher than that of people who do not participate in the research." Therefore, according to the regulations of the Department of Health Medical No. 1010265075, it is exempted from the ethics review committee after evaluation.

## Author contributions

S-CY: Writing – review & editing, Writing – original draft, Investigation, Data curation, Conceptualization. L-CH: Writing – review & editing, Data curation, Conceptualization.
